# Can clinical factors be determinants of bone metastases in non-small cell lung cancer?

**DOI:** 10.4103/1817-1737.30355

**Published:** 2007

**Authors:** Ahmet Ursavas, Mehmet Karadag, Esra Uzaslan, Erkan Rodoplu, Ezgi Demirdögen, Basak Burgazlioglu, R. Oktay Gozu

**Affiliations:** *Department of Pulmonary Medicine, School of Medicine, University of Uludag, Bursa, Turkey*

**Keywords:** Bone metastases, bone scanning, non-small cell lung cancer

## Abstract

**OBJECTIVE:**

The aim of this study was to investigate the correlations among symptoms, laboratory findings of bone metastasis and whole body bone scanning (WBBS) and the frequency of occurrence of bone metastases

**MATERIALS AND METHODS:**

Hundred and six patients who were diagnosed with non-small cell lung cancer (NSCLC) between June 2001 and September 2005 were investigated retrospectively. Bone pain, detection of bone tenderness on physical examination, hypercalcemia and increased serum alkaline phosphatase were accepted clinical factors of bone metastases. Presence of multiple asymmetric lesions in WBBS was also accepted as bone metastases. Subjects whose clinical factors and WBBS indicated doubtful bone metastases were evaluated with magnetic resonance and/or biopsy.

**RESULTS:**

Occurrence of bone metastases was 31.1% among all patients. Bone metastases were determined in 21 (52.5%) of 40 patients who had at least one clinical factor. Asymptomatic bone metastases without any clinical factors were established in 11.3% of all NSCLC patients and 15.3% of 26 operable patients. Sensitivity, specificity, positive predictive value, negative predictive value and accuracy of the clinical factors of bone metastases were 63.6, 73.9, 52.5, 81.8 and 70.7% respectively. There was no significant relationship between histologic type and bone metastases. Sensitivity, specificity, positive predictive value, negative predictive value and accuracy of WBBS in detection of bone metastases were 96.9, 86.3, 76.2, 98.4, 89.6% respectively.

**CONCLUSION:**

Sensitivity and specificity of the clinical factors of bone metastases are quite low. Routine WBBS prevented futile thoracotomies. Therefore, routine WBBS should be performed in all NSCLC patients, even in the absence of bone-specific clinical factors.

Lung cancer is the most common cause of cancer-related deaths throughout the world. Although the general incidence of cancer remains stable, there is a 0.5% increase in the global frequency of lung cancer.[1] The most important prognostic factor in non-small cell lung cancer (NSCLC) is tumor staging. Five-year survival rate in T1N0 local early stage lung cancer is 60-80%.[2] However, distant metastases are found in approximately 40% of patients with newly diagnosed lung cancer. Brain, liver, adrenal glands and bone are the most common sites of metastatic disease in NSCLC.[3] There is a tendency for futile thoracotomy, resulting in unsuccessful treatments and death, particularly in cases of NSCLC with asymptomatic distant metastases.

In the early stage (T1-2 N0) NSCLC, recurrence following surgical treatment is in the form of distant metastases (72%), and the most common metastatic sites are bone and the brain.[4] Bone metastasis frequently presents itself with findings such as pain, pathologic fractures, hypercalcemia and high levels of serum alkaline phosphatase. Therefore, performing a whole body bone scanning (WBBS) is still controversial in NSCLC patients with no clinical and laboratory findings associated with bone metastases. American Thoracic Society (ATS) and European Respiratory Society (ERS) do not recommend routine evaluation of distant metastases in patients with no symptoms and signs of metastases,[5] whereas the guidelines of American Collage of Chest Physicians (ACCP) and American Society of Clinical Oncology (ASCO) recommend routine evaluation of distant metastases only in cases of stage IIIA and IIIB.[6,7]

The aim of this study was to investigate the correlation between clinical factors of bone metastases and WBBS and the frequency of occurrence of bone metastases, as well as to evaluate necessity of WBBS in NSCLC.

## Materials and Methods

### Patients

Hundred and six patients (90 males, 16 females) who were diagnosed with non-small cell lung cancer between June 2001 and September 2005 at the pulmonary clinic of university hospital were investigated retrospectively. Patients previously diagnosed extrathoracic metastasis of NSCLC or any other cancer and those who died prior to the performance of WBBS were excluded from the study.

### Study design

In our unit, all patients diagnosed with NSCLC are routinely scanned for extrathoracic metastases. Local thoracic staging of all patients was done according to ‘International System for Staging Lung Cancer adopted by the American Joint Committee on Cancer and the International Union Against Cancer in 1997’,[2] using fiberoptic bronchoscopy and computed tomography, inclusive of the adrenals and liver. Lymph nodes with a short axis longer than 1 cm were accepted as pathological. Nodules outside the lobe of the primary tumor were classified as M1. Patients were divided two main groups after local intrathoracic staging. The first group was considered operable and consisted of T1-2-3 and radiologic N0-1 patients. Patients with radiologic N2-3, T4 made up the second, the unfavorable group.

^99m^Tc WBBS was performed in all patients included in the study. Presence of multiple asymmetric lesions in WBBS was accepted as bone metastases. Lack of any lesions or presence of a benign cause explaining the lesions observed in WBBS was accepted as normal; except for such lesions, all other lesions were considered to be doubtful. Bone pain, detection of bone tenderness on physical examination, hypercalcemia and increased serum alkaline phosphatase were accepted as clinical features suggestive of bone metastases. Subjects whose clinical factors and WBBS indicated doubtful bone metastases were evaluated with magnetic resonance (MR) and/or biopsy.

### Statistical analysis

Statistical analysis was performed using the SPSS package for Windows, version 13.0, and Chi-square and Fischer's exact tests at the Biostatistics Department. In addition, specificity, sensitivity, positive predictive value, negative predictive value and accuracy rates of clinical factors and WBBS in determining bone metastases were analyzed. A ‘*P*’ value less than 0.05 was considered statistically significant.

## Results

### Age, gender and histologic types

Bone metastases were detected in 33 (31.1%) of the subjects through WBBS, MR or biopsy. No significant differences were determined in the mean age and gender of subjects with or without bone metastases. Histologic types of cases were 67 (63.2%) squamous cell carcinomas, 24 (22.6%) adenocarcinomas, 2 (1.9%) large cell carcinomas and 13 (12.2%) unclassified non-small cell carcinomas. There was no significant difference in the incidence of histologic types between subjects with and without bone metastases.

### Symptoms and clinical factors of bone metastases

**Figure 1:**
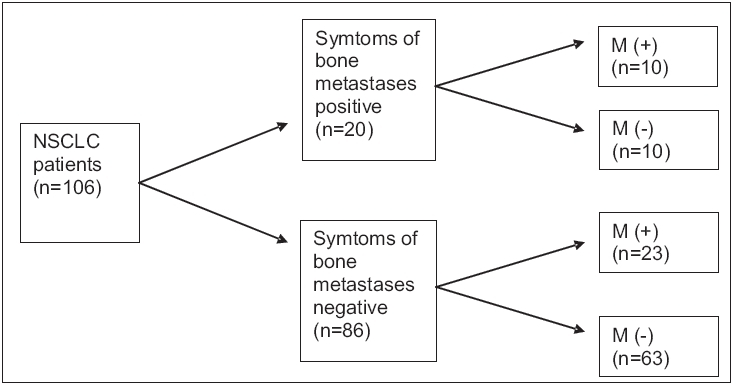
Symptoms of bone metastases

Bone metastases were present in 50% of patients who had the symptoms of bone metastases and in 26.7% of patients without any symptoms of bone metastasis [Figure 1]. Sensitivity, specificity, positive predictive value, negative predictive value and accuracy of symptoms alone in demonstrating bone metastases were determined to be 30.3, 86.3, 50, 73.2 and 68.9% respectively [Table 1].

**Table 1:** Accuracy rates of symptoms, clinical factors and whole body bone scanning in bone metastases

	Sensitivity %	Spesifisity %	PPV %	NPV %	Accuracy %
Symptoms	30.3	86.3	50.0	73.2	68.9
Clinical factors	63.6	73.9	52.5	81.8	70.7
Whole body bone scanning	96.9	86.3	76.2	98.4	89.6

**Figure 2:**
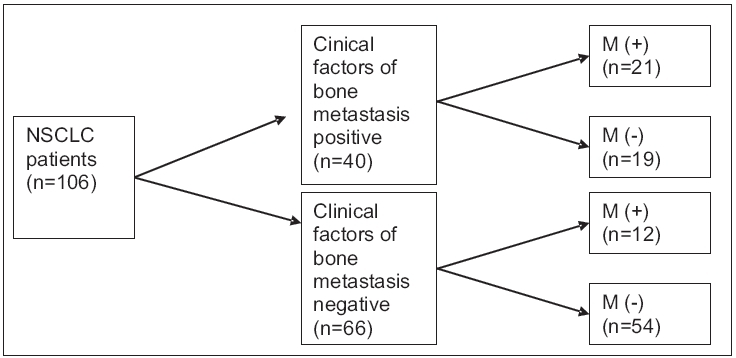
Clinical factors of bone metastases

Forty (34.9%) of the patients had at least one clinical factor (symptom, physical examination or laboratory findings) of bone metastases. Bone metastases were determined in 52.5% of patients who had at least one clinical factor. At the end of all investigations, incidence of asymptomatic bone metastases without any clinical factors was established in 11.3% of all NSCLC patients [Figure 2]. Sensitivity, specificity, positive predictive value, negative predictive value and accuracy of the clinical factors of bone metastases were 63.6, 73.9, 52.5, 81.8 and 70.7% respectively [Table 1].

### WBBS

There were abnormal findings of WBBS in 42 (39.6%) of the 106 patients. It was demonstrated that no metastasis was present in subjects whose bone WBBS revealed doubtful results or findings of WBBS and clinical factors were inconsistent - through MR (7 patients) and bone biopsy (3 patients). Sensitivity, specificity, positive predictive value, negative predictive value and accuracy of WBBS were determined to be 96.9, 86.3, 76.2, 98.4 and 89.6% respectively [Table 1].

According to thoracic CT, 26 patients were identified as N0-1 – probable, operable; and 80 patients (75.5%) as T4 and/or N2-3 – inoperable. Clinical factors of bone metastasis were present in 10 of 26 (38.4%) ‘probable, operable’ patients according to thoracic staging. Bone metastases were determined in 4 of patients who demonstrated clinical factors of bone metastases and 4 of 26 patients who were ‘probable, operable’ but demonstrated no clinical factors of bone metastasis.

**Figure 3:**
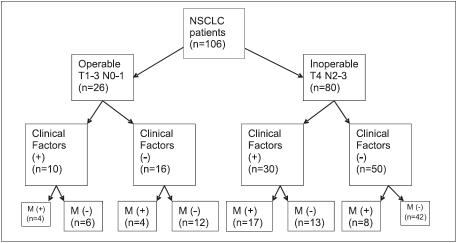
Clinical factors and bone metastases in operable and inoperable NSCLC

Twenty-two patients were identified operable according to ATS and ERS guidelines. Consequently, asymptomatic bone metastases without any clinical factors were established in 4 (15.3%) of the 26 operable patients. Bone metastases were determined in 8 (10%) of 80 inoperable patients who were asymptomatic and had no positive clinical factors [Figure 3].

## Discussion

Despite all the improvements in NSCLC therapy, relapse rates after curative surgical procedures remain to be very high. Majority of the relapses are determined in extrathoracic organs.[8] Accurately staging after diagnosis of NSCLC is important in preventing unnecessary thoracotomies. The incidence of distant metastases in lung cancer is high. There are symptoms, abnormal physical examination findings or laboratory findings in most of the bone metastases. It has also been reported that there may be asymptomatic metastases in patients with NSCLC.[9–12]

Bone, brain, adrenal glands and liver are the most frequent sites of metastasis. BTS recommends that computed tomography of the thorax should include liver and adrenal glands.[13] This method enables scanning of liver and adrenal glands without any delays or any increases in costs. However, the necessity of obtaining routine WBBS and cranial MR in patients without any signs of metastases remains to be controversial.

Bone metastases are seen in 10-20% of patients with lung cancer.[14] It is usually manifested with the symptom of pain. However, pain is a nonspecific symptom. Pain in patients with lung cancer may be due to other causes, just as there may be no pain in patients with metastasis to bone. Hetzel *et al.*[15] determined pain in 91 of 121 subjects with lung cancer; however, they identified bone metastases only in 40 (33%) of these patients and reported that the sensitivity of symptoms in identifying bone metastases was 53%. In this present study, we detected bone metastasis in 33 of 106 (31.1%) NSCLC patients. The symptom of pain was present in only 10 of our subjects, and we identified the sensitivity of this symptom to determine bone metastases as 30.3%. Besides the symptom of pain, there may also be tenderness on palpation during physical examination, and findings such as high calcium and alkaline phosphatase levels might be seen in routine laboratory examinations in bone metastases. Although Quinn *et al.*[16] reported to find at least one clinical sign of bone metastases in 33 of 53 NSCLC subjects, only 7 of these (21.2%) actually had bone metastases. However, Erturan *et al.*[17] have reported that they determined clinical signs of bone metastases in 39 of 125 subjects with NSCLC and bone metastases in 21 (53.8%) of these. In the present study, there were metastases to bone in 52.5% of subjects with clinical signs of bone metastases.

Metastases that are found in subjects who have no clinical signs or laboratory findings of metastases are termed as ‘silent metastasis.’ The rate of silent metastases to bone in NSCLC has been reported to be 2.5-30.3% in different series.[9,10,15–20] However, the important point is the presence of silent metastases in patients evaluated as operable according to thoracic staging, because skipping the scan for metastasis results in unnecessary thoracotomy in these patients. A controlled randomized multicentered study performed on 634 operable NSCLC subjects in Canada has reported that routine scanning for metastases prevents performance of unnecessary thoracotomies and is more cost-effective.[21] Asymptomatic bone metastases has been reported in 8% of T1-3 N0–1 operable NSCLC patients.[17] Extrathoracic metastases were determined by scanning in 24 of 95 NSCLC patients who were assessed as potentially operable with thorax CT, and 8 of these (8.4%) were reported to have bone metastases.[22] In our study, the rate of silent metastases in all subjects was 18.1%; however, this rate was 15.3% for our 26 subjects who were considered as operable according to thoracic staging (T1-3 N0-1).

It has been suggested that there might be an association between the stage of TN and cell type and metastases in lung cancer. Extrathoracic metastases were reported to be seen more frequently in adenocarcinomas than in squamous cell carcinomas in some studies.[19,23] However, there are also publications suggesting the idea that there is no association between cell types and TN stage and extrathoracic metastases.[18,19] In our study no significant association was identified between the stage of TN and cell type and the frequency of bone metastases.

WBBS is an effective method in determining bone metastases, but it may give doubtful or false positive results due to benign reasons such as trauma, surgery and osteoarthritis. In such cases, evaluation with methods including direct WBBS, MR and bone biopsy is required, which delays treatment and causes loss of time and money. The rate of false positivity in WBBS has been reported to be in between 20 and 40%.[16–20] In our study, there were real metastases to bone in 32 of 42 subjects in whom pathologies were determined in bone WBBS. The rate of false positivity was determined as 23.8%.

The 18-flourodeoxyglocose positron emission tomography (FDG-PET) method has been reported to be an effective method in evaluating mediastinal nodes and distant metastases in NSCLC.[24] The FDG-PET both shows asymptomatic bone metastases and has lower risk of false positivity.[25] At the onset of the evaluation period of subjects of this retrospective study (2001-2003), PET was not available in our country.

American Thoracic Society (ATS) and European Respiratory Society (ERS) do not recommend routine bone and brain scanning in NSCLC subjects who do not demonstrate any symptoms or clinical signs of metastases.[5] On the other hand, ACCP does not recommend routine scanning for metastases in asymptomatic subjects with stage I-II NSCLC and reports that routine scanning shall be performed only in stage III subjects.[6] Obtaining routine WBBS is not recommended by these treatment guides because of the high negative predictive values of bone-specific clinical factors and high false positivity rates of WBBS. However, numerous studies, particularly the study of Canada oncology group, have reported that routine scanning for extrathoracic metastases prevent unnecessary thoracotomies.[15–23]

## Conclusion

As our study was designed to be a retrospective method, information about the symptoms of bone metastases may be limited. Despite this, it is obvious that reliability of the symptoms and laboratory findings of bone metastases are very low. The rate of bone metastases that are asymptomatic and do not demonstrate any laboratory signs is quite high in NSCLC, including operable patients. Routine WBBS prevented four futile thoracotomies in 22 of our operable patients who according to ATS and ERS guidelines. According to the results of this study, we suggest that WBBS shall be obtained in all NSCLC patients.
